# Michaelis-Menten kinetics of soil respiration feedbacks to nitrogen deposition and climate change in subtropical forests

**DOI:** 10.1038/s41598-017-01941-8

**Published:** 2017-05-11

**Authors:** Jennifer Eberwein, Weijun Shen, G. Darrel Jenerette

**Affiliations:** 1 Department of Botany and Plant Sciences, University of California, Riverside, USA; 2 Key Laboratory of Vegetation Restoration and Management for Degraded Ecosystems, South China Botanical Gardens, Chinese Academy of Sciences, Beijing, China

## Abstract

China experiences some of the highest rates of anthropogenic nitrogen deposition globally, with further increases projected. Understanding of soil feedbacks to the combined anthropogenic influences of climate change and nitrogen deposition in these systems is critical to improve predictive abilities for future climate scenarios. Here we used a Michaelis-Menten substrate-based kinetics framework to explore how soil CO_2_ production (R_soil_) responds to changes in temperature and available soil nitrogen (N) by combining field experiments with laboratory manipulations from sites experiencing elevated rates of anthropogenic N deposition but varying in soil N availabiltiy. The temperature sensitivity of R_soil_ was strongly influenced by labile C additions. Furthermore, estimation of the temperature response of the Michaelis-Menten parameters supports the use of substrate-based kinetics in modeling efforts. Results from both field and laboratory experiments demonstrated a general decrease in R_soil_ with increasing soil available N that was variably dependent on carbon (C) availability. Both the field and the laboratory measurements demonstrated a consistent decrease in the Michaelis-Menten parameter kM with increasing soil available N, indicating an increase in the efficiency of soil C decomposition with increasing N. Furthermore, these results provide evidence of interactions between N deposition and temperature sensitivity, which could influence C storage under combined anthropogenic global change drivers.

## Introduction

Developing a mechanistic and predictive understanding of soil carbon (C) pool responses to interactive global change drivers remains an important research goal^1, 2^. In many regions soil C dynamics are influenced simultaneously by increased climate warming and nitrogen (N) deposition. With strong theoretical justification from thermodynamics and enzymatic processes^3, 4^, warming should lead to increased rates of soil CO_2_ production (soil respiration; R_soil_), however, a broad range of responses have been observed^5^. The effects of N enrichment are even more complex with conflicting theoretical predictions and empirical evidence showing increases, decreases and no response of R_soil_ to increased N availability^6–10^. The interactions between warming and nitrogen availability on soil C emissions are likely equally complex and may create a positive climate change feedback in a future warming climate. Resolving these uncertainties in R_soil_ responses to both warming and N availability is important for defining soil functioning and the repercussions of multiple interacting global change drivers.

Substrate-based mechanisms for describing soil biogeochemical processes can be a valuable tool for modeling and predicting soil feedbacks to anthropogenic global change drivers^11, 12^. This approach is hierarchical in that the effects of warming and nitrogen availability are conceptualized as influencing parameters in a substrate dependent model. A commonly-used model of substrate-based kinetics is the Michaelis-Menten model, which describes a saturating function of substrate concentration, with parameters V_max_, the maximum reaction velocity, and kM, the half-saturation constant, which corresponds to the substrate concentration [S] when V_max_/2, as follows:

1 $$R=\frac{{V}_{{\rm{\max }}}\ast [S]}{kM+[S]}.$$

The role of substrate-based limitation has been considered for assessments of R_soil_ temperature sensitivity, but there are competing hypotheses^13^. The inverse carbon-quality hypothesis predicts that low quality, recalcitrant substrates will require higher activation energy, and therefore be more temperature sensitive than high quality, labile substrates^14, 15^. Alternatively, the availability of C may be a greater regulator of the temperature sensitivity of R_soil_ via the temperature sensitivities of the Michaelis-Menten parameters, V_max_ and kM. If both are influenced by temperature this can create a canceling effect at low substrate concentrations when the importance of the kM temperature sensitivity is greater^11, 16^. Therefore, it is unclear whether carbon quality or availability is more important in regulation of the temperature sensitivity of R_soil_. Furthermore, few studies have empirically tested the temperature sensitivity of V_max_ and kM in soil^17, 18^.

How soil biogeochemical processes are influenced by N deposition, and the divergent responses of CO_2_ emissions to N availability are also yet to be resolved^19^. Through an N limitation perspective, relief of N limitation should increase soil microbial activity and lead to a positive response of soil CO_2_ emission. However, this cannot explain the suppressive influence of N on soil CO_2_ emission seen in many studies^6, 20, 21^. Alternatively, the N mining hypothesis describes the process where microbes “mine” recalcitrant carbon for N^22^. Through this process, increasing N alleviates the need for mining of recalcitrant C, thereby decreasing R_soil_. Another promising hypothesis incorporates the divergent response of N addition on R_soil_ through dynamic carbon-use efficiency (CUE) of soil microorganisms in order to adjust metabolic activity to better fit inconsistent resource availability^23–25^. This hypothesis predicts that the influence of N availability on R_soil_ is dependent on the stoichiometry between C and N. When C is limiting compared to N, microbes will increase their CUE, thereby decreasing R_soil_. Alternatively, when C is abundant compared to N, decreased CUE results in increased R_soil_. When both resources are abundant, metabolic activity and growth are at a maximum. Initial laboratory tests have demonstrated R_soil_ dynamics consistent with dynamic CUE in arid and semi-arid environments^9, 18^, but not in a mesic system or from field experiments.

In addressing this uncertainty, we examined how soils from a subtropical moist forest in Guangdong Province, China respond to increasing soil N availability. This region in the highly industrialized Pearl River Delta has experienced rapid land use change and the urbanized areas, including Guangzhou, have become a large source of N deposition to the outlying forest ecosystems^26^. China experiences some of the highest rates of N deposition globally, with further increases projected^27^. The selected study sites along an urban to rural transect allow for the unique opportunity to study soil respiration under high rates of anthropogenic N deposition (>30 kg N ha^−1^ y^−1^, see Table 1), but with varying amounts of soil available N, which we use as a proxy for increasing N saturation. Additionally, experimental N addition plots were used to compare differences in soil available N while holding other plant community and soil characteristics constant. We conducted field and laboratory experiments to evaluate how nitrogen deposition may influence R_soil_ in this region and potential interactions with warming. We asked: (1) How does soil N availability influence the response of R_soil_ to labile C addition? (2) How does labile C addition affect the temperature sensitivity of soil respiration, and (3) Does soil N availability influence this temperature response? We hypothesized that N availability would influence R_soil_ based on C:N stoichiometry, following the dynamic CUE hypothesis, and that temperature sensitivity would be mediated through the Michaelis-Menten parameters, V_max_ and kM.

**Table 1:** Site characteristics.

Site	Lat/Long	MAT (C)/MAP (mm)	N deposition (kg N ha^−1^ y^−1^)	Soil pH	Dominant tree species	Tree ht (m)	DBH (cm)
Pougang^26, 42^ (urban)	E113°48′, N23°37′	21.5/1700	41.2 ± 6.5	3.71 ± 0.1	*Schima superba*, *Ardisia quinquegona*, *Castanopsis chinensis*, *Psychotia rubra*, *and Lophatherum gracile*.	*14*.*0* ± 3.0	*17*.*3* ± 4.0
Luogang^26, 42^ (suburban)	E113°18′, N23°06′	21.9/1738	35.2 ± 7.8	3.80 ± 0.2	*Schima superba*, *Cryptocarya concinna*, *Diplospora dubia*, *Castanopsis chinensis*, *and Machilus chinensis*	*15*.*0* ± 4.0	*17*.*9* ± 7.1
Shimentai^43, 44^ (rural)	E113°05′, N24°22′	20.8/1700	34.1 ± 6.4	3.55 ± 0.01	*Schima superba*, *Cryptocarya concinna*, *Machilus chinesis*, *Castenea henryi (Skan*) Rehd., and *Engelhardtia roxburghiana*	*13*.*8* ± 2.5	*18*.*6* ± 5.2
Heshan^40, 45^	E112°50′, N22°30′	22.5/1534	43.1 ± 3.9	3.83 ± 0.03	*Acacia auriculiformis*, *Schima superba*, *Michelia macclurei*, *Cinnamonum camphora*, *Magnoliaceae glance*	*13*.*2* ± *2*.*7*	*16*.*0* ± *6*.*3*

Values are mean  ± 1 standard deviation.

## Results

### Soil N

**Figure 1:**
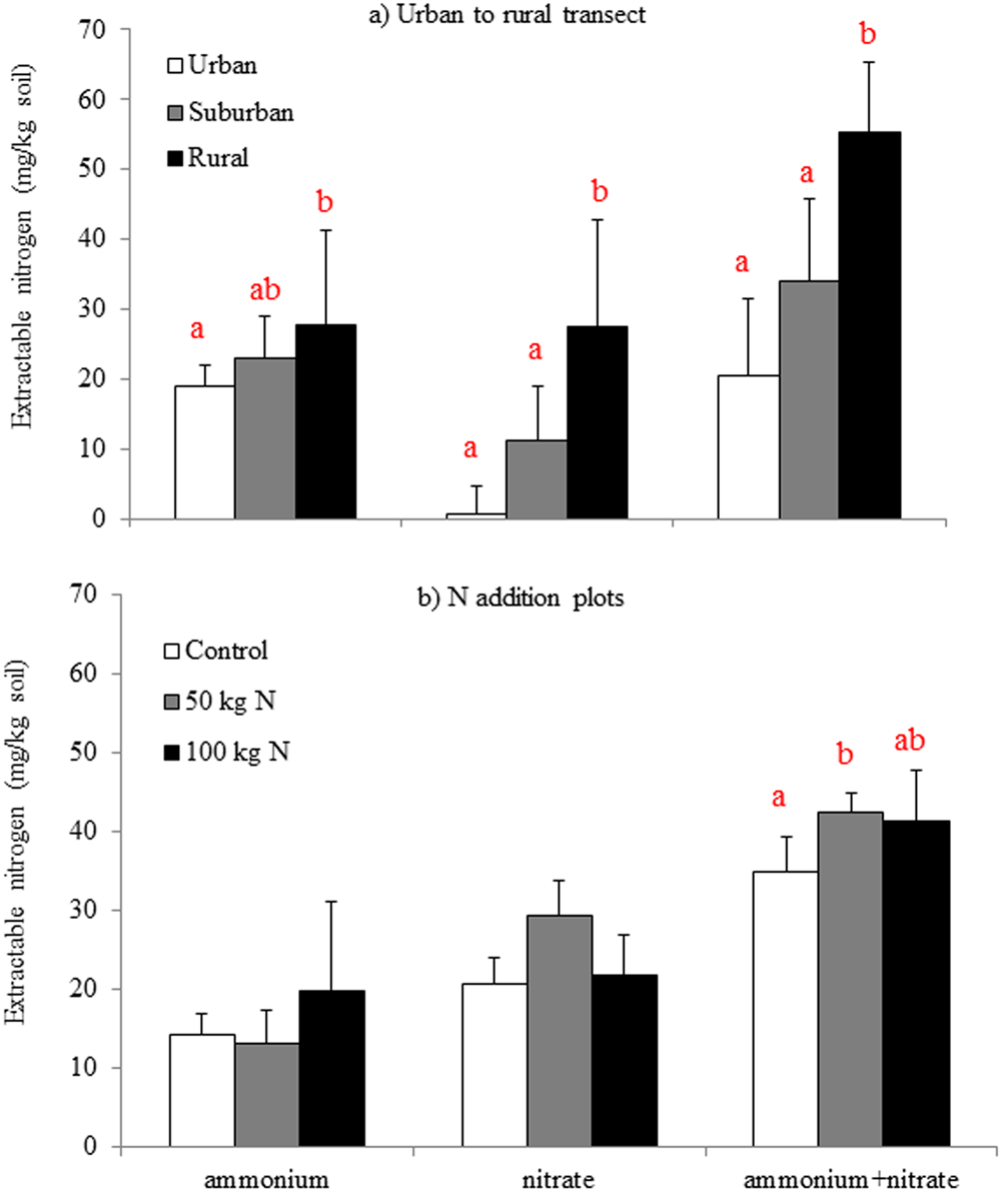
Soil KCl extractable nitrogen in the urban to rural transect (**a**) and the nitrogen addition plots (**b**). Error bars represent standard deviation. Letters represent significance at p < 0.05 from 1-way ANOVA followed by Tukey HSD (n = 5 for a and n = 3 for (**b**)).

Soils from the urban to rural transect sites spanned a range of available N (NH_4_^+^ plus NO_3_^−^) from 14.7 to 71.4 mg kg^−1^ (Fig. 1a). While total N availability was significantly different (p < 0.05) between the rural and urban sites and the rural and suburban sites, the urban and suburban sites were not significantly different. For simplicity, the urban site will hereafter be referred to as “Low N”, the suburban site as “Mid N” and the rural site as “High N”. The Heshan Station N addition plots ranged from 30.3 to 49.6 mg N kg^−1^ soil (Fig. 1b). While there were no significant differences between plots for ammonium or nitrate individually, total N for the control plot was significantly less than the 50 kg N ha^−1^ y^−1^ plot, but not significantly different from the plot receiving 100 kg N ha^−1^ y^−1^ plot (at p < 0.05). To maintain consistency, the N addition plots have been relabeled to reflect differences in soil available N, similar to the urban to rural transect. The plots receiving 50 kg N ha^−1^ y^−1^ presented the highest soil total available N and will therefore be referred to as “High N”, the plots receiving 100 kg N ha^−1^ y^−1^ will be referred to as “Mid N”, and the control plots will be referred to as “Low N”.

### N influence on R_soil_

**Figure 2:**
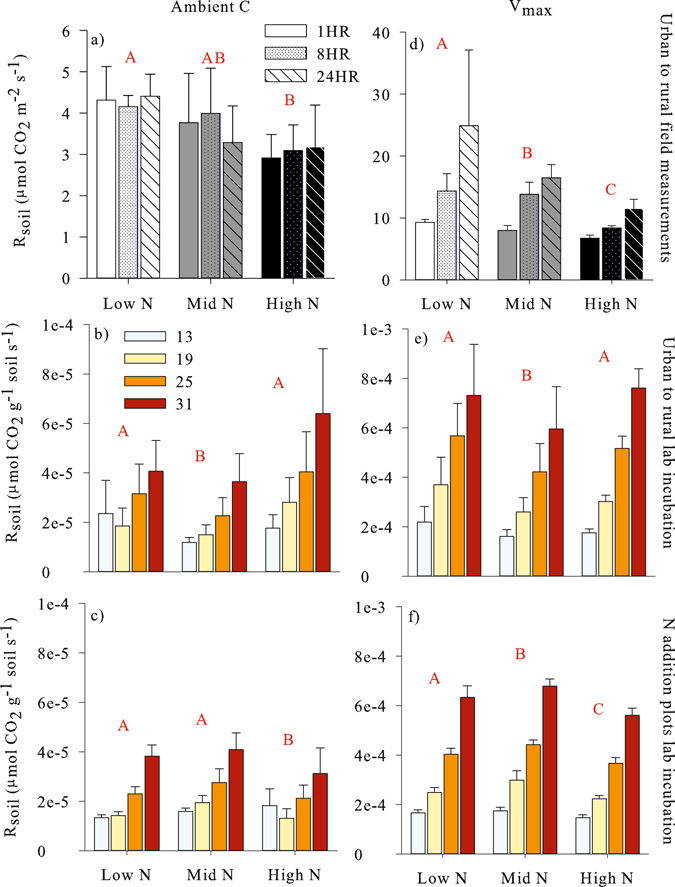
Soil CO_2_ production (R_soil_) for ambient soil carbon (**a**–**c**) compared to saturating carbon (V_max_; (**d**–**f**)) for the field measurements along the urban to rural transect (**a**,**d**), the laboratory incubation of soils from the urban to rural transect (**b**,**e**), and the laboratory incubation of soils from the nitrogen addition plots. Legends indicate time since treatment addition (**a**,**d**) or temperature in °C (**b**,**c**,**e**,**f**). Error bars represent standard deviation. Letters represent significance at p < 0.05 from mixed-linear model followed by Holms-corrected Tukey Contrasts for a and d (n = 4) and 2-way ANOVA followed by Tukey HSD for (**b**,**e**) (n = 5) and (**c**,**f**) (n = 3).

For all measurements, soil CO_2_ production generally decreased with increasing soil extractable N levels (Fig. 2). Field measurements along the urban-to-rural transect showed decreasing CO_2_ production with increasing soil available N (Fig. 2a,d; p < 0.05). The laboratory incubation results for the urban to rural transect (Fig. 2b,e) showed decreased CO_2_ production in the Mid N soils compared to the Low N soils at both ambient and saturating C concentrations (p = 0.04 and 0.0004, respectively). However, CO_2_ production in the High N soils was not significantly different from the low N soils (p = 0.2 and 0.4 for ambient and V_max_, respectively). In the N addition plots (Fig. 2c,f), CO_2_ production was decreased in the High N soils compared to the Low N soils (p < 0.0001 and p = 0.003 for ambient and V_max_, respectively). In the urban to rural transect, the response of R_soil_ to soil N was the same at both ambient soil C concentrations (Fig. 2a,b) and at saturating C concentrations (V_max_; Fig. 2d,e). However, in the N addition plots, the response at saturating C concentrations was different from ambient C concentrations. While there was no difference between the Low N and Mid N soils at ambient concentrations (p = 0.1), there was an increase in V_max_ from Low N to Mid N (p = 0.006).

### Michaelis-Menten parameters

**Figure 3:**
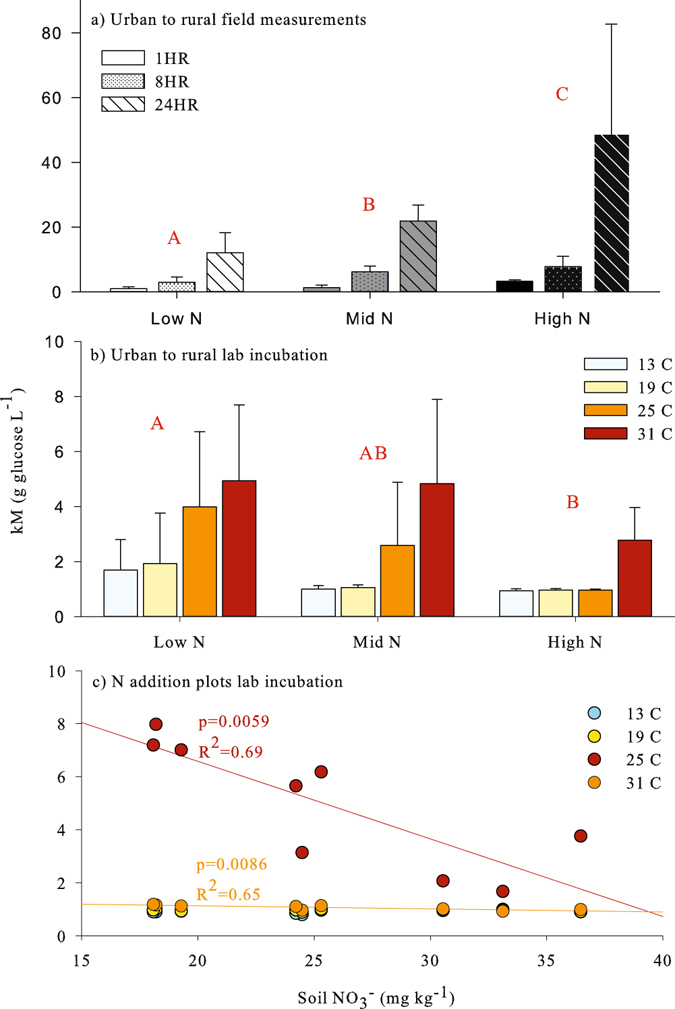
The Michaelis-Menten half-saturation constant (kM) for the field measurements along the urban to rural transect (**a**), the laboratory incubation of soils from the urban to rural transect (**b**), and the laboratory incubation of soils from the nitrogen addition plots (**c**). Legends indicate time since treatment addition (**a**) or temperature in °C (**b**). Error bars represent standard deviation. Letters represent significance at p < 0.05 from mixed-linear model followed by Holms-corrected Tukey Contrasts for a (n = 4) and 2-way ANOVA followed by Tukey HSD for (**b**) (n = 5). In (**c**) lines represent significant linear regressions.

The Michaelis-Menten parameter kM also demonstrated a general pattern of decline with increasing available N across all soils (Fig. 3). Results of the field experiment for the urban to rural transect demonstrated the decrease in R_soil_ with increasing soil N, evident at all sampling times (Fig. 3a). R_soil_ was measured at 1, 8 and 24 hours after glucose additions for fitting the Michaelis-Menten parameters V_max_ and kM, which both increased with time since wetting (Figs 2d and 3a). The laboratory incubation experiment with the soils from the urban to rural transect further allowed for manipulation of temperature to understand the temperature sensitivity of kM. Generally, kM declined from the low N to the high N soils across all temperatures (Fig. 3b). Furthermore, in the laboratory incubation of soils from the N addition plots, kM was negatively related with measured soil N (Fig. 3c) at 25 and 31 °C (p < 0.05). Also apparent from the temperature manipulations, kM was highly temperature dependent and this relationship was non-linear.

### Temperature sensitivity of R_soil_

**Figure 4:**
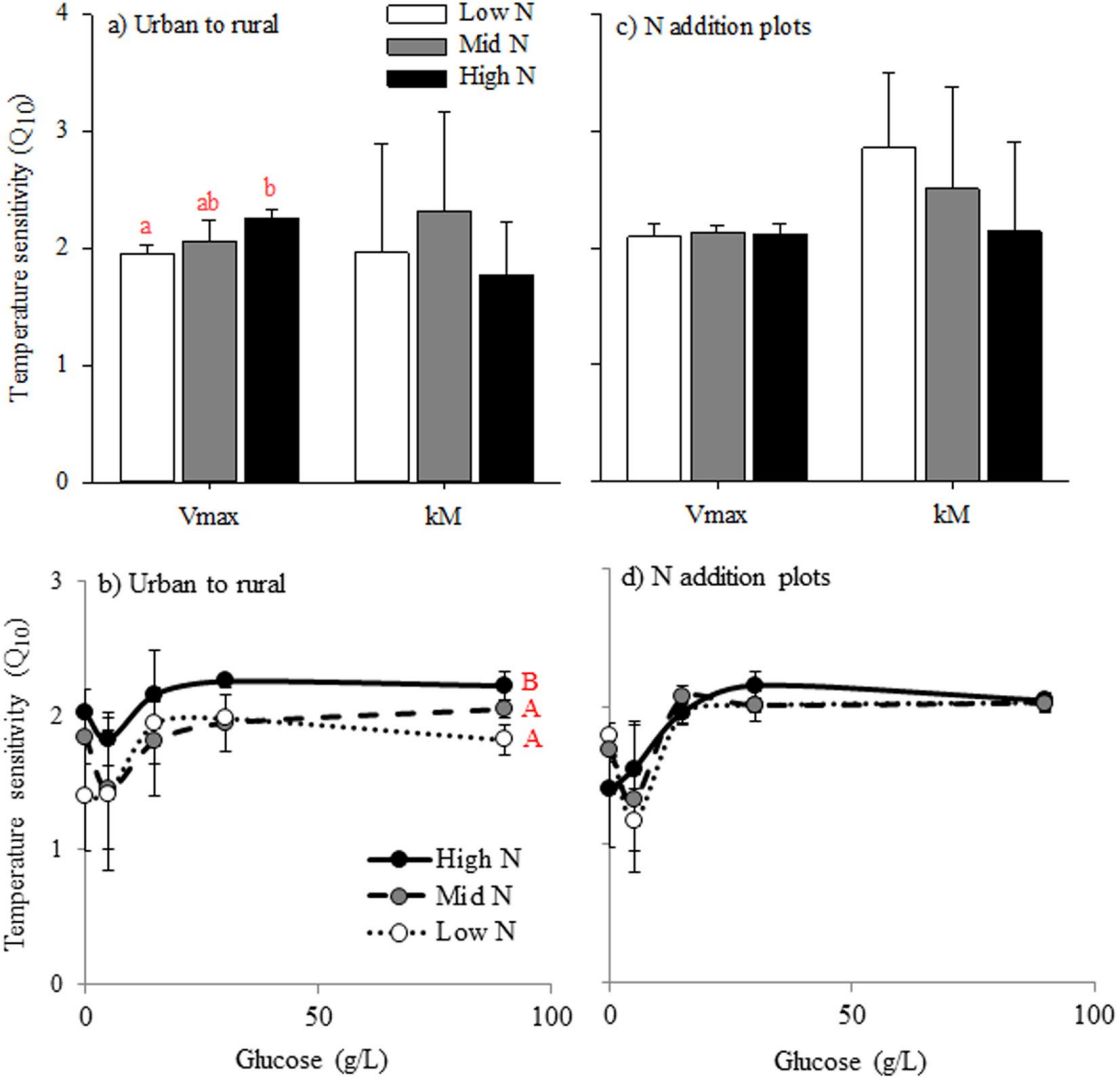
Temperature sensitivity (Q_10_) of the Michaelis-Menten parameters V_max_ and kM (**a**,**c**), and Q10 versus glucose addition (**b**,**d**). Error bars represent standard deviation. Letters represent significant difference at p < 0.05 from one-way (**a**,**c**) or 2-way (**b**,**d**) ANOVA followed by Tukey HSD (n = 5 for (**a**,**b**) and n = 3 for (**b**,**d**)).

Additionally, the laboratory incubations allowed for exploration of relationships between availability of labile carbon and the temperature sensitivity of R_soil_ (Fig. 4). The incubation results from the urban to rural transect soils showed a significant increase in the Q_10_ of V_max_ with increasing N (p < 0.05), but no relationship between soil N and the Q_10_ of kM (Fig. 4a). N also increased R_soil_ Q_10_ in the high N compared to the low and mid N treatments for all C concentrations (Fig. 4b). Alternatively, soils from the N addition plots did not exhibit differences in Q_10_ of V_max_ or kM (Fig. 4c). For both the urban to rural transect and the N addition plots, there was a divergent response of R_soil_ Q10 to glucose addition, with a general decrease from ambient carbon to 5 g/L glucose, followed by an asymptotic increase from 5 g/L through 90 g/L glucose (Fig. 4b,d).

## Discussion

Our findings demonstrate that in mesic subtropical forests complex interactions among C availability, warming, and N availability influence R_soil_, a key ecosystem process with potential climate feedbacks. Our findings provide initial experimental support for modeling assumptions based on coupled temperature and substrate dependent R_soil_ kinetics^11, 16^. Further, our study provides experimental results of decreasing R_soil_ with increasing N availability consistent with general global patterns in forest soils^8, 10, 21^ and a dynamic CUE mechanism for the response of R_soil_ to N availability^18, 28^.

First-order decay of C input is a fundamental property of the C cycle that is shared across all ecosystems, presenting a valuable tool for modeling global terrestrial C budgets^29^. Substrate-based kinetics allow for a mechanistic approach to predict changes in the first-order decay rates of R_soil_ in response to warming^11^. Here we show the first experimental estimation of R_soil_ Michaelis-Menten parameters for decomposition of a labile C source in whole soil. The laboratory incubations demonstrated positive temperature sensitivity for both V_max_ and kM (Fig. 4), which indicate a non-linear relationship with temperature similar to Arrhenius kinetics. This positive temperature response of both V_max_ and kM confirms the potential for a canceling effect at low substrate concentrations^5^. However, the C availability influence on temperature sensitivity produced a divergent response in almost every soil. Compared to the control treatment that received no labile C addition, there was a decrease in Q_10_ with initial C addition and then an increase with subsequent increasing C addition (Fig. 4b,d), which indicates that both the C quality and the C availability hypotheses are working together. While using a general enzymatic approach to represent R_soil_ necessarily simplifies the complex metabolic pathways and physical structure of the soil, results from this study demonstrate that these theoretical simplifications for modeling efforts are useful for whole soils and in field conditions. Such an integrated metabolic framework suggests a pathway to improve soil C modeling and predictions of soil C storage under future climate and nitrogen pollution scenarios.

A key uncertainty in C substrate and temperature sensitivity models is the additional inclusion of the effects from other resources. With globally increasing rates of N availability, an evaluation of a potential N effect on R_soil_ may be important for predicting soil climate feedbacks. As found across previous studies^6, 18, 30^, we likewise observed variable influences of N availability. In general N availability reduced R_soil_ emissions at both ambient and saturating substrate concentrations, consistent with the nitrogen mining hypothesis (Fig. 2). However, we observed some evidence of N limitation that, when alleviated, increased R_soil_ capacity. Our findings were consistent with the variable CUE hypothesis, which predicts increasing N leads to greater conservation of available C through decreased respiratory loss^28^. Furthermore, in the N addition plots, differences in V_max_ associated with available soil N resulted in greater V_max_ in the Mid N soil compared to the control, but lower V_max_ in the High N plot compared to the control (Fig. 2f). This pattern corresponds to a shift from N limitation in the low N soil, to balanced C:N availability, and then C limitation in the highest available N soil, consistent with shifts in CUE regulating R_soil_ as previously observed from laboratory incubations from dryland ecosystems^18, 31^ and that we extend to tests from more mesic environments and through the use of field experiments.

While our results demonstrated a general decrease in R_soil_ with increasing N availability, differences between field results and lab measurements may reflect the influence of autotrophic respiration. Some studies have demonstrated a disproportionate influence of N on soil root and mycorrhizal respiration compared to heterotrophic respiration^10, 21, 32^. This would explain the strong response in the field experiments, but a less consistent response in lab incubations. Furthermore, in the lab incubation for the N addition plots (Fig. 2f) the divergent response of V_max_ to N availability would reflect the isolation of heterotrophic respiration, highlighting the importance of microbial CUE, which was obscured by the autotrophic response in the field results. This heterotrophic response might be harder to discern in the urban to rural transect where it is impossible to maintain absolute consistency in plant community composition. However, overall there was a consistent decrease in R_soil_ with increasing soil N availability across all study systems.

Across all experiments in this study, increasing soil N resulted in a reduction in the Michaelis-Menten parameter, kM (Fig. 3) and we interpret this as a further indicator of dynamic CUE influence on R_soil_. In traditional enzyme assays, kM represents the inverse of an enzyme’s affinity for its substrate^33, 34^. In the context of this study, kM allows for an estimation of efficiency as it represents the balance between CO_2_ production for a particular amount of substrate addition. The decrease in kM suggests a more efficient use of labile C with increasing N, contrary to the expected view for CUE. However, application of kM to CUE in this study would require that microbial biomass and extracellular enzyme production remain constant across treatments as CO_2_ production was the only parameter measured. While we only measured CO_2_ production, many studies have shown decreases in microbial biomass with N additions^8, 30, 35–37^. Alternatively, the empirical kM estimates from this study could be interpreted in the more traditional sense as a shift in enzyme specificity. N addition can result in shifts in extracellular enzyme production that are more targeted at C decomposition and therefore more efficient rather than enzymes directed at both C and N acquisition^34, 38^. Regardless, the consistent decrease in kM with increasing soil N availability across all soils in this study presents a useful tool for modeling R_soil_ dynamics under anthropogenic nitrogen deposition.

Importantly, we also found that N availability can influence the sensitivity of R_soil_ to both temperature and C substrate availability, presenting a potential for interaction between N deposition and climate change. N addition caused a significant increase in the temperature sensitivity of R_soil_ in the urban to rural transect (Fig. 4a,b). The N influence on temperature sensitivity of the Michaelis-Menten parameters was limited to V_max_ Q_10_ and not kM Q_10_. This could result in a dampening of the canceling effect and therefore greater temperature sensitivity at lower substrate concentrations under N deposition, which is supported by results from the urban to rural transect demonstrating greater Q_10_ at the high N site (Fig. 4b). Furthermore, the influence of N on kM in the N addition plots was magnified at higher temperatures. A decrease in kM would result in an increase in R_soil_ at less than saturating substrate concentrations. As C is usually not saturating in most natural settings the decrease in V_max_ could be of less significance than the decrease in kM in terms of annual C emissions.

In conclusion, our results provide needed experimental support for theoretical relationships between R_soil_, temperature and N, and highlight the benefit of substrate-based kinetics for improving understanding of soil biogeochemical processes, particularly R_soil_ temperature and N sensitivity. Although increasing soil N can decrease R_soil_, interactions between N and temperature could offset this response and result in greater loss of soil C stores. Anthropogenic activities have increased N inputs to terrestrial systems by about 46 Tg N y^−1^, with about one third being deposited to forests^39^. Developing a mechanistic understanding of soil feedbacks to the combined anthropogenic influences of climate change and nitrogen deposition in these systems is critical to develop predictive abilities for future climate scenarios, and substrate-based kinetics provide a valuable tool to help accomplish that goal.

## Methods

### Study Sites

The study was located in Guangdong Province of South China. The area is under the influence of a subtropical monsoon climate with alternating wet and dry seasons, and has undergone rapid urban expansion since the 1970s. Three sites were selected along an urban to rural transect in Guangdong Province in southern China to capture an anthropogenic N deposition gradient^26^. The urban to rural transect started in the urban site of Pu Gang in the South China Botanic Gardens, then the suburban site of Lou Gang, and ended in the Shimentai Nature Reserve in Yingde County. All sites were located in late successional evergreen broadleaf forests with at least 50 years since disturbance. All soils were latosolic red. Published nitrogen deposition data from these sites reveal higher than expected nitrogen deposition in the rural site (Table 1). Increased canopy interception of N deposition in urban sites may explain the gradient in soil available N seen in this study^26^ (Fig. 1).

Additionally, soils were collected from N addition plots in a mixed legume and native forest at Heshan National Field Research Station of Forest Ecosystems in Heshan County, Guangdong Province. The plots consisted of three replicates each of a control (no additions), medium N addition (50 kg N ha^−1^ yr^−1^), and high N addition (100 kg N ha^−1^ y^−1^) for a total of nine plots. N was applied as ammonium nitrate in 10 L of water with a backpack sprayer once a month for two years prior to analysis^40^. Additional site characteristics are summarized in Table 1.

### Field measurements

Plots were established at the three sites along the urban to rural transect to compare *in situ* R_soil_ responses to glucose additions. For these experiments, soil collars measuring 20 cm in diameter and 5 cm in height were set 24 hours before sampling in a plot measuring approximately 25 m^2^ at each site along the transect. Treatments included a control (water only) and four levels of glucose concentration: 5, 15, 30 and 90 g/L. Each treatment was replicated four times for a total of 20 samples per site. 200 mL of water or glucose solution were applied to each collar, and soil respiration was measured with a portable infrared gas analyzer (LI-8100, Licor Biosciences) at 1, 8 and 24 hours after addition of water or glucose solution.

### Incubation experiments

Soils were collected from the three sites along the urban to rural transect as well as the nine N addition plots at Heshan Station to perform laboratory incubations to explore the response of soil respiration to glucose addition under controlled temperature and moisture conditions. Five field replicates were collected from each plot. Each field replicate was composed of approximately ten soil cores collected from 0–15 cm of the mineral soil layer. The soils were brought back to the lab and air dried for 3 to 5 days at room temperature. The soils were then sieved to 2 mm and homogenized. Before drying, an aliquot from each field replicate was placed in the freezer for nitrate and ammonium analysis. An additional aliquot from the dried and sieved samples was used for total organic C and total N analysis. Soil chemical analysis was performed by the Key Laboratory of Vegetation Restoration and Management for Degraded Ecosystems at the South China Botanical Gardens, Chinese Academy of Sciences using the copper cadmium reduction-diazotization coupling method for nitrate, potassium chloride leaching-indophenol blue method for ammonium, Kjeldahl method for total nitrogen and external heating using potassium dichromate oxidation method for total organic carbon^41^. Water holding capacity (100%) was determined by the gravimetric water content of soil placed in a filter funnel and saturated with deionized water, then allowed to drain for two hours.

The same five treatments were applied to the incubation experiments as were used in the field: control (water only), 5, 15, 30 and 90 g/L of glucose. In total, the three sites along the urban to rural transect included five treatments and five field replicates for a total of seventy-five samples. For the N addition plots at Heshan station, there were five glucose treatments, three N levels, and three replicates (one for each plot) for a total of forty-five samples. For each replicate, 50 g of soil was placed in a 200 ml Erlenmeyer flask with a vented rubber stopper. The glucose solutions (and water in the case of the control) were used to bring the soils to 40% WHC, and then they were incubated at 25 °C for 24 hours. After the incubation period, soil respiration was measured with an infrared gas analyzer (LI-6262 Licor Biosciences) at 13, 19, 25, and 31 °C. Fluxes were calculated from the linear portion of the curve generally between 30 and 90 seconds after the jar was sealed, and adjusted for chamber volume, soil weight and chamber temperature. The CO_2_ flux measurements were then fit to the Michaelis-Menten model with nonlinear least squares regression to determine V_max_ and kM using Matlab (2015b). Temperature sensitivity (Q10) was determined by the following equation:

2 $$Q10=R2/R1{}^{\tfrac{10}{{T}_{2}-{T}_{1}},}$$

where R1 and R2 are soil respiration parameters (R_soil_, V_max_ or kM where applicable) measured at temperatures T1 and T2, respectively.

### Statistical Analysis

Statistical significance for treatment effects for the laboratory incubations were determined by 2- and 3-way Analysis of Variance (ANOVA) using the (R version 3.2.5). Assumptions of normality and homoscedacity were tested with the Shapiro-Wilks and Bartlett’s tests, respectively. When these assumptions were not met, the Box-Cox family of transformations was used. Analysis of field measurements used linear mixed-effects model by maximum likelihood with time as a random factor followed by Tukey Contrasts with Bonferroni-Holm correction for multiple comparisons using the lme4 and multcomp packages in R version 3.3.3. Linear regressions for the relationship between kM and available soil nitrogen were performed in Matlab (2015b).

### Data availability statement

The datasets generated during and/or analyzed during the current study are available from the corresponding author on reasonable request.
